# A Multi-Dimensional Calibration Based on Genetic Algorithm in a 12-Bit 750 MS/s Pipelined ADC

**DOI:** 10.3390/mi14091738

**Published:** 2023-09-05

**Authors:** Hanbo Jia, Xuan Guo, Huaiyu Zhai, Feitong Wu, Yuzhen Zhang, Dandan Wang, Kai Sun, Danyu Wu, Xinyu Liu

**Affiliations:** 1 Institute of Microelectronics of the Chinese Academy of Sciences, Beijing 100029, China; jiahanbo@ime.ac.cn (H.J.); zhaihuaiyu@ime.ac.cn (H.Z.); wufeitong@ime.ac.cn (F.W.); zhangyuzhen@ime.ac.cn (Y.Z.); wangdandan1@ime.ac.cn (D.W.); sunkai1@ime.ac.cn (K.S.); wudanyu@ime.ac.cn (D.W.); 2 University of Chinese Academy of Sciences, Beijing 100049, China

**Keywords:** analog-to-digital converter, pipelined, capacitor mismatch, interstage gain error, genetic algorithm

## Abstract

As the preferred architecture for high-speed and high-resolution analog-to-digital converters (ADC), the accuracy of pipelined ADC is limited mainly by various errors arising from multiple digital-to-analog converters (MDAC). This paper presents a multi-dimensional (M-D) MDAC calibration based on a genetic algorithm (GA) in a 12-bit 750 MS/s pipelined ADC. The proposed M-D MDAC compensation model enables capacitor mismatch and static interstage gain error (IGE) compensation on the chip and prepares for subsequent background calibration based on a pseudo-random number (PN) injection to achieve accurate compensation for dynamic IGE. An M-D coefficient extraction scheme based on GA is also proposed to extract the required compensation coefficients of the foreground calibration, which avoids falling into local traps through MATLAB. The above calibration scheme has been verified in a prototype 12-bit 750 MS/s pipelined ADC. The measurement results show that the signal-to-noise and distortion ratio (SNDR) and spurious-free dynamic range (SFDR) are increased from 49.9 dB/66.7 dB to 59.6 dB/77.5 dB with the proposed calibration at 25 °C. With the help of background calibration at 85 °C, the SNDR and SFDR are improved by 3.4 dB and 8.8 dB, respectively.

## 1. Introduction

Since [1] proposed the concept of redundant bits and [2] implemented the 1.5-bit/stage structure, comparator requirements in pipelined analog-to-digital converters (ADC) have significantly relaxed. Since then, pipelined ADC has evolved from just a concept to the preferred structure of high-speed and high-resolution ADC [3,4]. With the rapid development of applications in wireless communication, high-end instrumentation and other fields, there is an increasing demand for higher accuracy of ADC [5,6,7]. As a result, accurately calibrating errors present in pipelined ADC has become a research focus for achieving improved overall accuracy [8,9,10,11,12,13,14].

**Figure 1:**
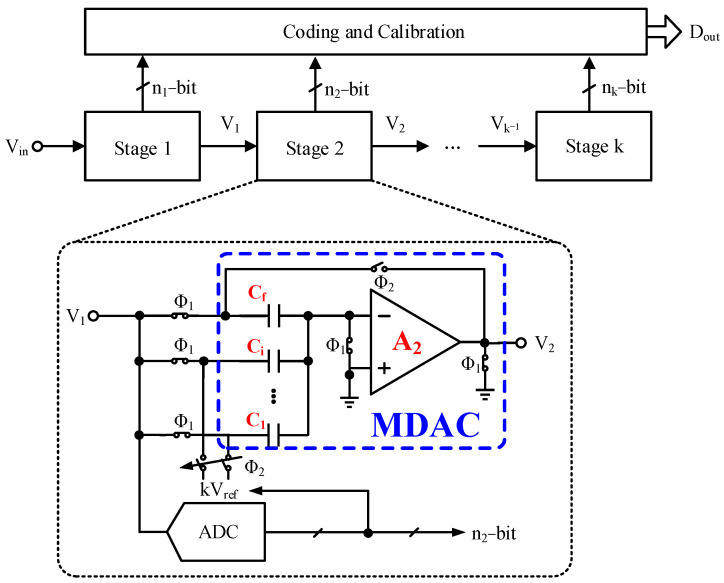
The basic architecture of a pipelined ADC.

Figure 1 shows the basic architecture of a pipelined ADC consisting of multiple pipelined stages that sequentially convert input signals into digital codes. Each stage mainly comprises a sub-ADC, sub-analog-to-digital converter (sub-DAC), subtractor and multiplier. The sub-DAC, subtractor and multiplier are commonly referred to as a multi-digital-to-analog converter (MDAC). As a very important component, the MDAC is the primary source of errors in pipelined ADC [15]. Firstly, a capacitor mismatch in the MDAC is inevitable due to limitations in integrated circuit manufacturing technology, leading to DAC errors and static interstage gain errors (IGE) [11]. These errors typically do not change with temperature and voltage changes due to capacitors being passive devices, enabling one-time calibration through the foreground methods. However, accurately obtaining corresponding compensation coefficients is a challenge. The operational amplifier (op-amp) is composed of MOSFETs, which are active devices, causing the gain of the operational amplifier to vary with temperature and voltage, resulting in dynamic errors in interstage gain. Therefore, real-time background calibration is necessary, because it does not need to interrupt the normal operation of the ADC [16,17]. The most common background calibration for dynamic IGE is pseudo-random number (PN) injection calibration, which uses the statistical characteristics of a PN to extract the MDAC’s interstage gain error [18,19,20]. Nevertheless, inaccurate PN injection compensation caused by capacitor mismatch can reduce ADC performance and result in inaccurate background calibration of dynamic IGE. 

At present, there are two common pipelined ADC capacitor mismatch calibration schemes. First, the weight of the flip capacitor is measured by manually controlling the output of the comparator in the analog domain and observing the change of the output code of the post-stage [15,21,22]. Although this method is simple and direct, it also has a series of problems. Measuring the bit weight of the corresponding capacitor needs to input a specific voltage, but the noise of the input signal will affect the measurement accuracy. At the same time, the redundant switch circuit will deteriorate the high-frequency performance of the overall ADC. The generation circuit of the specific voltage will also cause additional power consumption and area. Secondly, the corresponding weight of the capacitor is estimated by directly searching the capacitor weight in the digital domain combined with relevant performance indicators. If the search is carried out in a traversal way, it requires a huge amount of computation to ensure the accuracy of the search, which is not meaningful. If the search step is variable, it may fall into the trap, and the weight value is a local optimal solution, which cannot achieve the best performance of the ADC. 

To address these issues, this paper presents a multi-dimensional (M-D) MDAC calibration for pipelined ADC. It can effectively calibrate the DAC error and IGE due to capacitor mismatch and finite op-amp gain. First, an M-D MDAC compensation model is proposed that can be used to compensate for capacitor mismatch and static IGE in the foreground calibration, while also being compatible with the compensation of a PN-injected capacitor mismatch, which is prepared for accurate compensation of dynamic IGE through subsequent background calibration. Additionally, an M-D coefficient extraction technique based on a genetic algorithm (GA) is presented. The customized GA is used to analyze the output data of the pipelined ADC through MATLAB, which can accurately and effectively extract the compensation coefficients required by the M-D MDAC compensation model. Then, the compensation coefficients are written into the on-chip eFuse for error compensation in the foreground calibration. The above calibration scheme was implemented in a 12-bit 750 MS/s pipelined ADC and verified.

This paper is organized as follows. Section 2 analyses the MDAC error and describes the calibration based on the coefficient and the existing problems of this method. Section 3 describes the proposed M-D MDAC compensation model and the GA used to obtain the calibration coefficients in detail. Section 4 comprises the test results. Section 5 offers a summary of the entire paper.

## 2. Calibration Based on Coefficient

### 2.1. MDAC Error Analysis

For pipelined ADC, after the problem of the sub-ADC comparator threshold was solved by redundant bit technology, the MDAC became the most important error source at the pipelined stage, mainly involving two errors: the DAC error and IGE. On the one hand, the reduction of the capacitor area leads to a decline in the matching accuracy under the advanced technology. On the other hand, the decline in the intrinsic gain of the transistor leads to a decline in the op-amp gain. The DAC error is related to the former, causing the MDAC output curve to drift at the corresponding sub-range. The IGE is affected by both, resulting in the overall slope change of the MDAC output curve.

**Figure 2:**
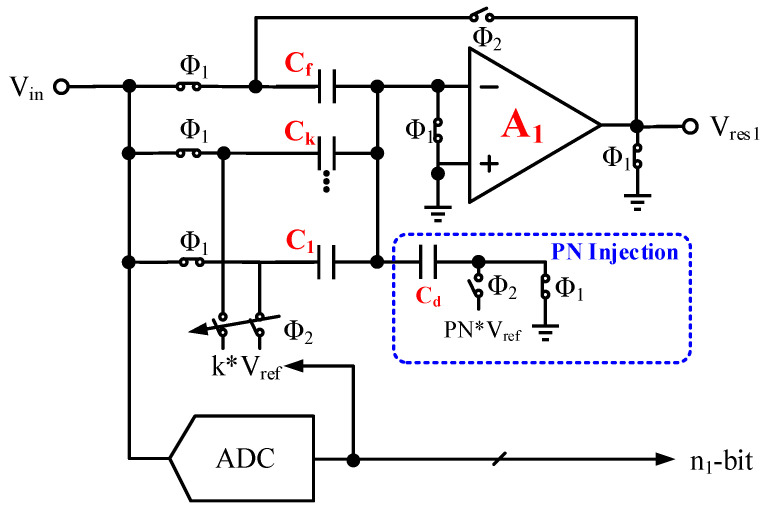
The MDAC architecture with PN injection.

**Figure 3:**
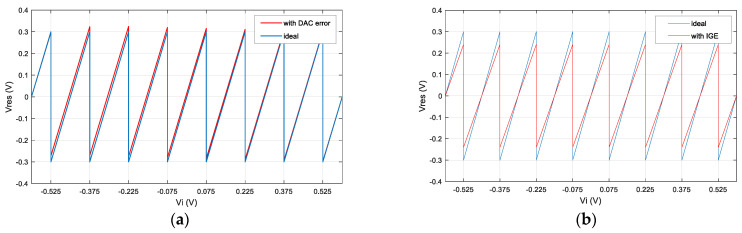
The influence of (**a**) DAC error and (**b**) IGE on the residual curve.

Here, a simplified capacitor-turnover MDAC model is used as an example for modeling in Figure 2. A two-level PN is injected into the MDAC in the first stage for calibration of the dynamic IGE. The residual voltage output by the first stage is:

(1) $$V_{res1}=\frac{1}{1+\frac{1}{A_{1}\cdot \beta }}\cdot \left\{\frac{\sum_{i}C_{s\_1}^{i}}{C_{f\_1}}\cdot V_{in}-\frac{\sum_{i}\left[C_{s\_1}^{i}\cdot D_{i}\cdot V_{ref}\right]+C_{d}\cdot D_{PN}\cdot V_{ref}}{C_{f\_1}}\right\}$$

where the feedback coefficient, $\beta =\frac{\sum_{i}C_{s\_1}^{i}+C_{d}+C_{f\_1}}{C_{f\_1}}$, $C_{s\_1}^{i}$, is the *i*-th sampling capacitor value of the first pipelined stage, $C_{f\_1}$ is the feedback capacitor value of the first pipelined stage, and $C_{d}$ is the PN injection capacitor value of the first pipelined stage. $D_{1}$ is the conversion digital code of the first pipelined stage, and $D_{PN}$ is the PN code injected into the first pipelined stage. The process deviation in a sampling capacitor will not only affect the residual voltage of the corresponding sub-range but also cause changes in the corresponding sub-region and higher amplitude sub-range, as shown in Figure 3a. 

The IGE can be decoupled into two parts, static IGE, $\frac{\sum_{i}C_{s\_1}^{i}}{C_{f\_1}}$, due to capacitor mismatch and dynamic IGE, $1+\frac{1}{A_{1}\cdot \beta }$, due to limited op-amp gain. A small interstage gain will lead to a lower slope of the residual curve, as shown in Figure 3b.

### 2.2. Compensation Based on Bit Weight 

The common compensation method of pipelined ADCs is based on the weight of the bit [21,23,24]. This can be summarized using the following expression:

(2) $$D_{o}=\sum_{i}D_{i}\cdot {\mathrm{ra}}_{s\_1}^{i}+D_{backend}-D_{PN}\cdot {\mathrm{ra}}_{d}$$

Here, $D_{o}$ is the overall output digital code, $D_{backend}$ is the post-stage converted digital code, $D_{PN}$ is the first-stage output digital code, ${\mathrm{ra}}_{s\_1}^{i}$ is the weight coefficient of the first-stage sampling capacitor, and ${\mathrm{ra}}_{d}$ is the weight coefficient of the first-stage PN injection capacitor. If the capacitor mismatch compensation based on the coefficient is carried out in the first three stages, $D_{backend}$ will be calculated as follows:

(3) $$D_{backend}=\sum_{i}D_{2}^{i}\cdot {\mathrm{ra}}_{s\_2}^{i}+\sum_{i}D_{3}^{i}\cdot {\mathrm{ra}}_{s\_3}^{i}+D_{res}$$

We can observe that the PN injection information and the input signal information are modified by the first-stage sampling capacitor weight coefficient, ${\mathrm{ra}}_{s\_2}^{i}$/${\mathrm{ra}}_{s\_3}^{i}$, which means that the compensation of the PN injection is affected by the capacitor mismatch in the post-stage. If the PN injection is not precisely compensated, it will lead to a deterioration in the signal-to-noise and distortion ratio (SNDR) and also impact the interstage gain estimation, ultimately leading to inaccurate background calibration.

There are two primary approaches for extracting the weight coefficient: analog and digital. In the analog approach, a manual control function is integrated into the comparator design [15,21,22]. Based on the specific voltage of the input, combined with manually controlling the output code of the comparator to control the flipping of the corresponding sampled capacitor, the weight of the flip capacitor can be measured by observing changes in the post-stage’s output code. However, the noise of the input signal will affect the measurement accuracy, while the redundant switch circuit will deteriorate the overall ADC’s high-frequency performance. Moreover, generating the specific voltage required will causes additional power consumption and area. In contrast, the digital way is to estimate the corresponding weight of the capacitor by combining the search method with relevant performance indicators. Although this method does not need to change the analog circuit, it requires a huge amount of computation if the search is carried out by traversal. If the search step is variable, it may fall into a trap.

## 3. M-D MDAC Calibration

### 3.1. M-D MDAC Compensation Model

The least mean square (LMS) algorithm based on the PN injection is widely used for background calibration of IGE. Injecting the PN into the MDAC will dither the subsequent pipelined stage. If the compensation for the capacitor mismatch in the post-stage is incomplete, it will also result in inaccuracies in compensating for the PN injection at this stage, leading to inaccurate background calibration. To accurately compensate for capacitor mismatch errors and IGE in the digital domain, a proper mathematical compensation model is essential. Simply transform (1) to obtain (4):

(4) $$V_{res1}=\frac{G_{1}}{f(A)}\cdot \left\{V_{in}-\frac{\sum_{i}\left[C_{s\_1}^{i}\cdot D_{1}^{{}_{i}}\cdot V_{ref}\right]+C_{d}\cdot D_{PN}\cdot V_{ref}}{\sum_{i}C_{s\_1}^{i}}\right\}$$

where $G_{1}=\frac{\sum_{i}C_{s\_1}^{i}}{C_{f\_1}}$, $f(A_{1})=1+\frac{1}{A_{1}\cdot \beta }$. Due to the mismatch of the process deviation capacitor, if the ideal bit weight is used for calculation, it will be inconsistent with the actual conversion and will significantly reduce the dynamic performance of the ADC. 

Taking into account the issues mentioned above, we can begin by setting the capacitor weight estimation coefficients, $\hat{C_{s\_1}^{i}}$ and $\hat{C_{d}}$, for the sampling capacitor mismatch and PN injection capacitor mismatch, respectively. We can then use $\hat{G_{1}}$ instead of $\frac{G_{1}}{f(A_{1})}$ as an estimation of the interstage gain. With these adjustments, the overall digital code obtained from the ADC can be expressed as:

(5) $$D_{o}=\sum_{i}D_{1}^{i}\cdot \hat{C_{s\_1}^{i}}\cdot \hat{G_{1}}+D_{backend}-\frac{\hat{C_{d}}}{\sum_{i}\hat{C_{s\_1}^{i}}}\cdot D_{PN}\cdot \hat{G_{1}}$$

where $\hat{G_{1}}=\frac{\sum_{i}\hat{C_{s\_1}^{i}}}{C_{f\_1}}$. In this way, the mismatch error of the feedback capacitor, $C_{f\_1}$, the static IGE and the dynamic IGE in the current state can be combined into $C_{s\_1}^{i}$. At this time, it is only necessary to find the appropriate capacitor weight estimation coefficient, $\hat{C_{s\_1}^{i}}$, to simultaneously compensate for the capacitor mismatch error and IGE in the current state, and also provide a convergence starting point for the compensation of the dynamic IGE, $\hat{G_{1}}$. If the gain in the op-amp varies with temperature and voltage, resulting in the change of interstage gain, the background calibration can be performed by modifying $\hat{G_{1}}$.

At the same time, considering the process mismatch construction of the PN injection capacitor, the weight estimation coefficient, $\hat{C_{d}}$, of the PN injection capacitor is obtained. $\hat{C_{s\_2}^{i}}$ and $\hat{C_{s\_3}^{i}}$ are related to $D_{2}$/$D_{3}$. However, due to the limited accuracy of $\hat{C_{s\_2}^{i}}$ and $\hat{C_{s\_3}^{i}}$, the residual errors of different $D_{2}$/$D_{3}$ combinations in the post-stage will affect the accurate compensation of the PN injection. In order to solve this problem, another PN injection noise compensation coefficient is designed, called $\alpha_{PN}$, to finely classify and compensate the PN injection. 

Theoretically, $\alpha_{PN}$ should include the combination of all the digital codes in the second/third pipelined stage that were selected by $D_{2}$/$D_{3}$, but $\alpha_{PN}$ will be an n × n vector. This complex model has no practical significance. Practically, $\hat{C_{s\_3}^{i}}$ has a small impact on the first pipelined stage PN compensation and can be ignored, and then $\alpha_{PN}$ will be an n × 1 vector and selected only by $D_{2}$.

**Figure 4:**
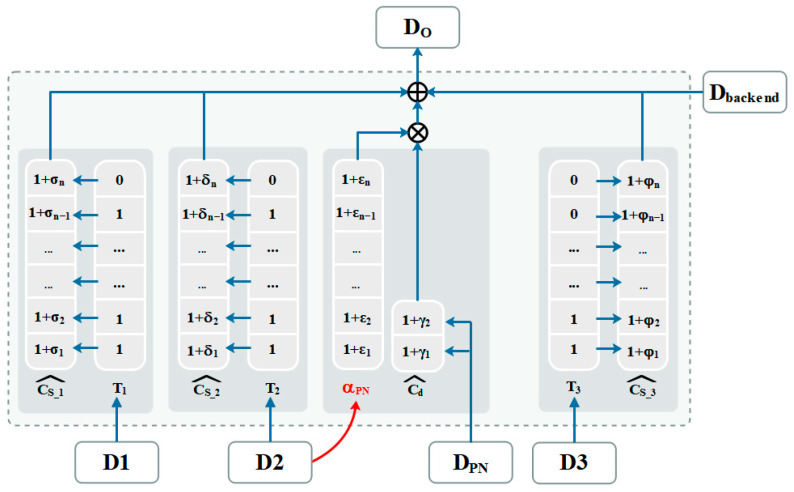
M-D MDAC compensation model block diagram.

**Figure 5:**
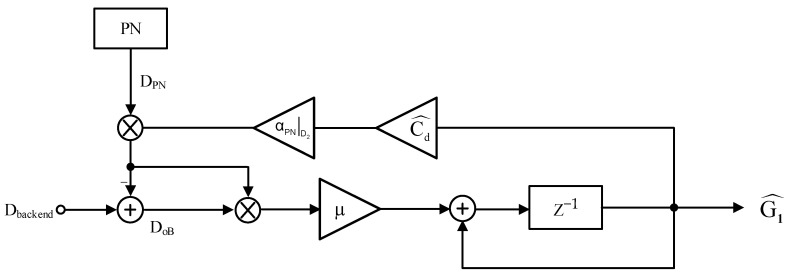
The block diagram of modified inter-stage gain error calibration block diagram based on the LMS algorithm.

Assuming that foreground calibration is implemented at the first three stages and the PN injection occurs at only the first stage, the compensation model block diagram is shown in Figure 4. $\hat{C_{s\_1}^{i}}$/$\hat{C_{s\_2}^{i}}$/$\hat{C_{s\_3}^{i}}$ achieve compensation for the capacitor mismatch and static IGE in the first three stages. Meanwhile, $\alpha_{PN}$ (controlled by $D_{2}$) and $\hat{C_{d}}$ (controlled by $D_{PN}$) are used together to achieve mismatch compensation for dither injection capacitor, $C_{d}$. Now, $D_{o}$ is calculated as follows:

(6) $$D_{o}=\sum_{j=1}^{3}\left[\sum_{i}D_{j}^{i}\cdot \hat{C_{s\_j}^{i}}\cdot \hat{G_{j}}\right]+D_{backend}-\hat{C_{d}}\cdot {\alpha_{PN}|}_{D_{2}}\cdot D_{PN}\cdot \hat{G_{1}}$$

where ${\alpha_{PN}|}_{D_{2}}$ represents the PN injection noise compensation coefficient when $D_{2}$ takes different values. The LMS iterative loop will be modified accordingly, based on the above coefficients:

(7) $$\hat{G_{1}}\left(n+1\right)=\hat{G_{1}}\left(n\right)+\mu \cdot D_{oB}\cdot D_{PN}$$

where $D_{oB}=D_{backend}-\hat{C_{d}}\cdot {\alpha_{PN}|}_{D_{2}}\cdot D_{PN}\cdot \hat{G_{1}}\left(n\right)$. The modified IGE calibration block diagram based on the LMS algorithm is shown in Figure 5. The digital code, $D_{backend}$, is obtained from the post-stage. The dither code, $D_{PN}$, is multiplied by $\hat{G_{1}}$/$\hat{C_{d}}$/${\alpha_{PN}|}_{D_{2}}$ and is subtracted from $D_{backend}$. The result is multiplied by $D_{PN}$ and μ, then passes through an accumulator to give an estimate for the inter-stage gain, $\hat{G_{1}}$. A large μ gives a fast convergence and low accuracy.

The proposed foreground calibration method utilizes the injection noise compensation coefficient, $\alpha_{PN}$, and the PN injection capacitor compensation coefficient, $\hat{C_{d}}$, to achieve accurate compensation and compatibility for the PN injection. This method also ensures effective operation of the background calibration, improving the overall system performance.

### 3.2. M-D Compensation Coefficient Extraction Based on GA

The calibration’s accuracy is dependent on the precision of the compensation coefficient extraction process. Extracting coefficients for the mentioned compensation can be viewed as an M-D optimization process. To obtain the most globally optimal solution, this paper introduces a novel approach by using GA to extract the compensation coefficients through MATLAB [25,26,27,28].

GA is a method of finding the optimal solution by simulating natural evolution processes. Compared to traditional optimization algorithms, GA has many advantages. The two most noteworthy are the ability to handle complex problems and parallelism. GA can deal with various types of optimizations, regardless of whether the objective (fitness) function is stationary or non-stationary, linear or nonlinear, continuous or discontinuous, with or without random noise. Due to the fact that multiple descendants in a population act like independent agents, the population (or any subpopulation) can simultaneously explore the search space in multiple directions, making it easy to parallelize. 

**Figure 6:**
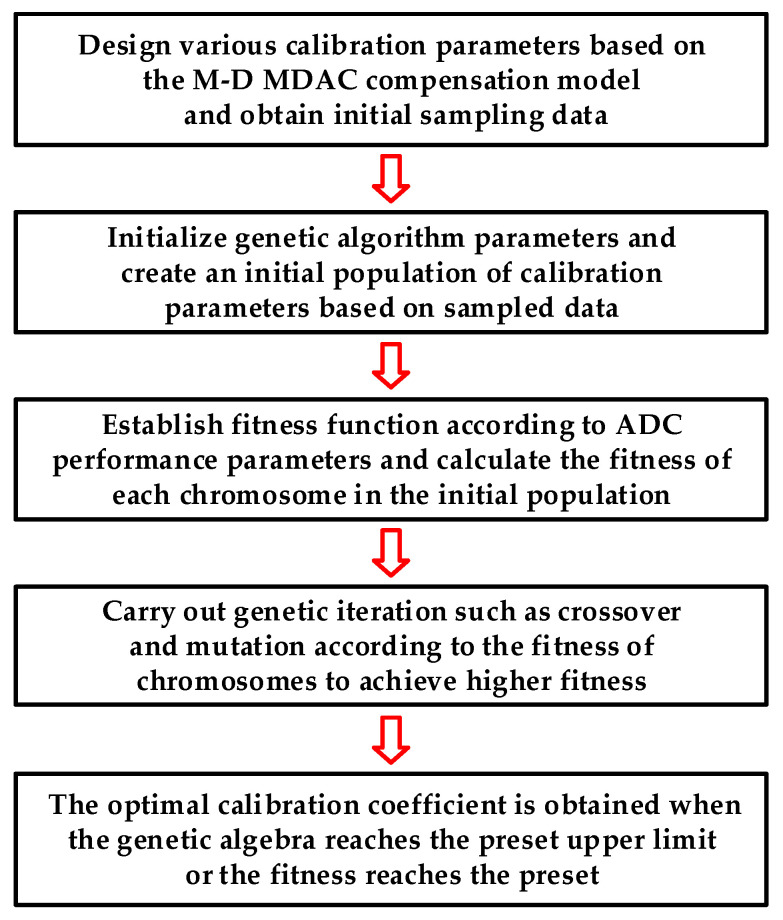
Flow diagram for extracting multi-dimensional compensation coefficients based on GA.

Figure 6 presents the flowchart for extracting M-D compensation coefficients utilizing the GA. First, obtain the initial data required for calibration and design various calibration parameters based on the proposed compensation model. Second, initialize the GA parameters and create an initial population of calibration parameters based on sampled data. Third, establish the fitness function and calculate the fitness of each chromosome in the initial population. Then, carry out genetic iterations such as crossover and mutation according to the fitness of the chromosomes to achieve higher fitness, until the genetic algebra reaches the preset upper limit or the fitness reaches the preset [26,29]. During the process of extracting compensation coefficients, the population initialization, fitness function establishment and population evolution are important steps that will be mainly introduced.

#### 3.2.1. Population Initialization

To calibrate the ADC, it is necessary to convert a section of the sine input signal into digital data first. Subsequently, based on the calibration compensation model mentioned above, the subsequent initialization process of the population can be conducted. The population initialization process comprises two stages: problem modeling and model mapping.

During the problem modeling stage, it is necessary to describe the optimization objectives in mathematical models. In this case, the three optimization coefficients, $\hat{C_{s}}$/$\hat{C_{d}}$/$\alpha_{PN}$ in the calibration compensation model mentioned above are the objectives to be optimized.

**Figure 7:**
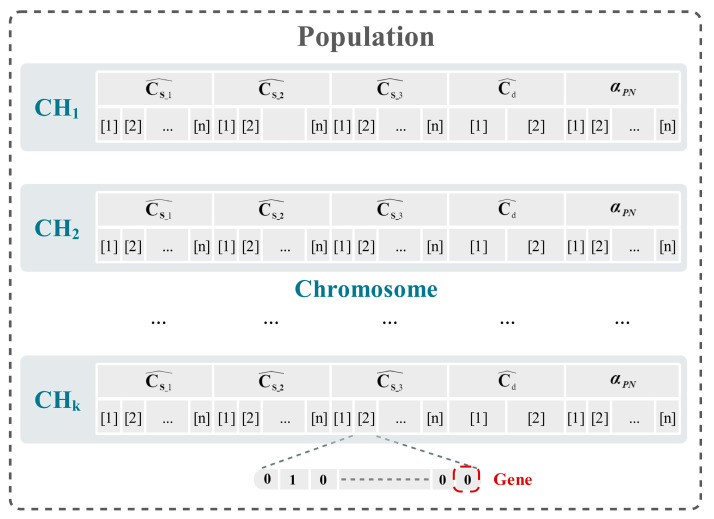
Coding forms and related concepts of calibration coefficients.

In the model mapping stage, the coefficients are mapped onto a specific coding form. This coding is referred to as a “chromosome”, where each single code point within the chromosome is called a “gene”, and the corresponding calibration model output is labeled as a “phenotype”. Specifically, the vector length of the calibration parameter $\hat{C_{s}}$/$\hat{C_{d}}$/$\alpha_{PN}$ is designed according to the structure, and the value range is set [30]. A set of random values within the range are then initialized. This set of random initial values represents a chromosome within the context of GA. The chromosomes are then expanded to form an initial population, as shown in Figure 7. Generally, the chromosomes should extend to cover the entire solution space as much as possible during the population initialization.

#### 3.2.2. Fitness Function 

To obtain performance parameters such as the effective number of bits (ENOB) and spurious-free dynamic range (SFDR), FFT is used to calculate the power spectrum estimation of each chromosome’s phenotype in the initial population through MATLAB. Through this process, the fitness function can evaluate the calibration effect of each calibration coefficient. ENOB is a better choice, as it includes all the information on the noise and distortion components, thereby reflecting the overall dynamic performance of ADC. 

In order to avoid falling into the local optimum called “precocity”, it is necessary to create a large enough initial population and ensure that the chromosomes have low similarity to cover the entire solution space as much as possible. The specific method is similar to the Hamming distance [31,32,33,34]. The difference between the chromosome representing the highest ENOB and the elements of other chromosomes is used to obtain the similarity. A chromosome set with sufficiently high ENOB and low similarity is then selected as the initial population for genetic evolution.

#### 3.2.3. Population Evolution

Population evolution is essentially a probabilistic operation, where the probability of chromosome selection is directly related to fitness. The goal here is to leave and multiply chromosomes with higher fitness. Selection, crossover and mutation are the three core operations that constitute the evolutionary process of GA.

**Figure 8:**
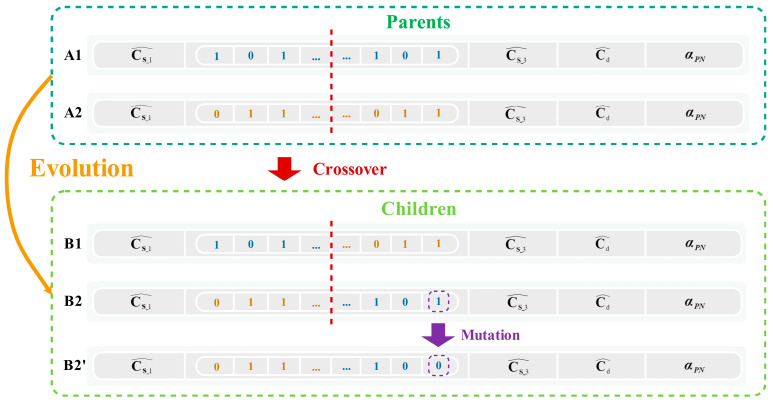
Crossover and mutation of calibration coefficients.

Single-point crossover involves exchanging one segment of a chromosome with the corresponding segment on another chromosome at random locations, as depicted in Figure 8. Crossover can also occur at multiple sites, which involves exchanging multiple segments with corresponding segments on their chromosomes. Multi-point crossover is often used to improve the evolutionary efficiency of the algorithm.

The mutation operation involves randomly flipping selected bits. To determine the optimal mutation rate, the mutation rate is designed as a function related to the optimal ENOB and the average ENOB of the population. The mutation rate is adaptively adjusted throughout the iteration, maintaining a high mutation rate at the beginning and gradually reducing it towards the end to ensure smooth convergence.

Finally, the optimal chromosome and its corresponding fitness value in each generation of evolution are saved as a basis for the convergence of the algorithm. After a sufficiently long period of genetic operation, a set of globally optimal calibration coefficients can be obtained.

## 4. Measurement Result

**Figure 9:**
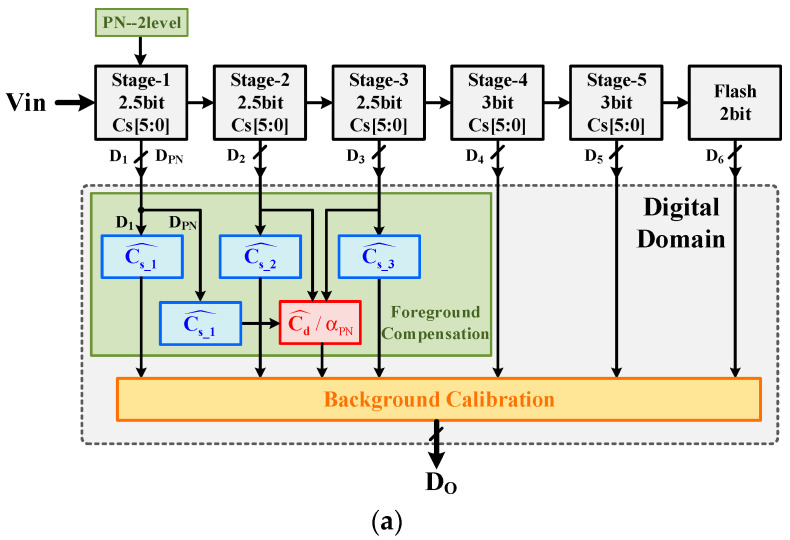

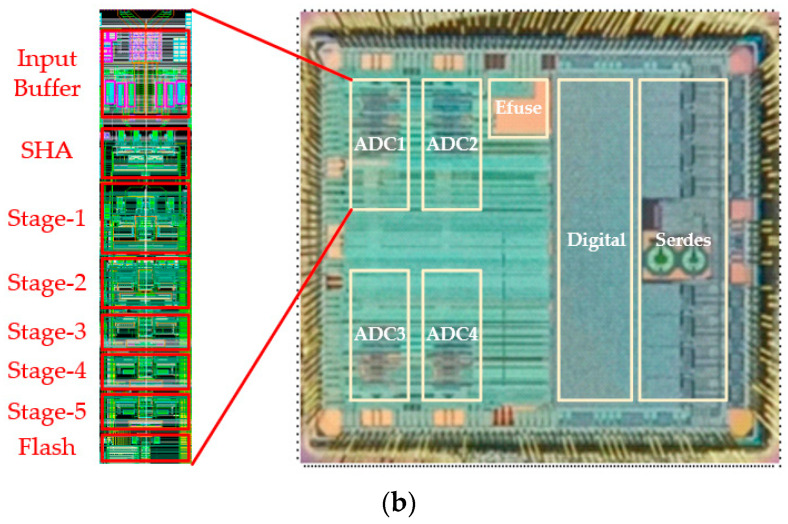
(**a**)The structure block diagram of this ADC and (**b**) the ADC micrograph.

To verify the calibration scheme proposed above, we applied it to a 12-bit 750 MS/s pipelined ADC. The block diagram of this ADC structure is shown in Figure 9a. Only the first three stages are calibrated in the foreground, while the first stage MDAC is injected with a two-level PN for background calibration. The ADC prototype is manufactured in a 40 nm CMOS process. The die micrograph is shown in Figure 9b, including four independent ADCs, a digital module, eFuse and SerDes output (for fewer pins about data output). The digital module on the chip mainly consists of the digital calibration part (foreground compensation and background calibration), the SPI interface and debugging registers, which facilitate control and debugging during the ADC testing. The chip occupies an area of 3 mm × 3 mm, and the digital calibration area is about 0.2 mm^2^ for a single ADC.

**Figure 10:**
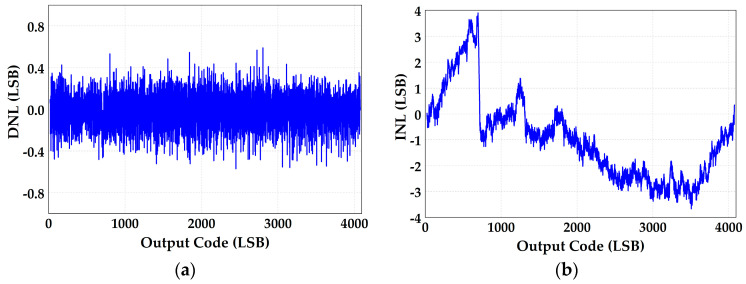
The static performance of (**a**) DNL and (**b**) INL without the proposed calibration.

**Figure 11:**
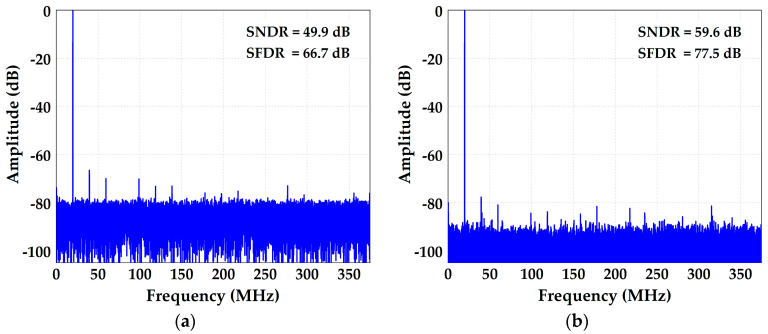
ADC spectral performance (**a**) without foreground calibration and (**b**) with the proposed calibration at 25 °C.

**Figure 12:**
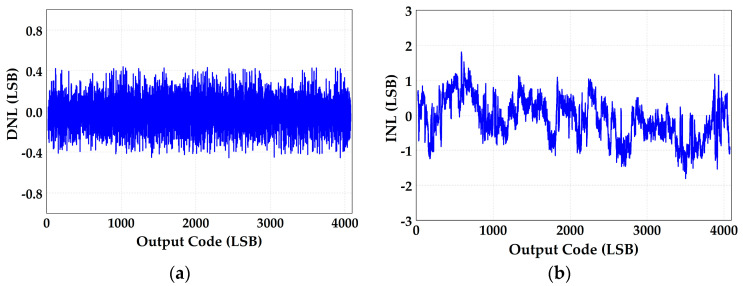
The static performance of (**a**) DNL and (**b**) INL with the proposed calibration.

**Figure 13:**
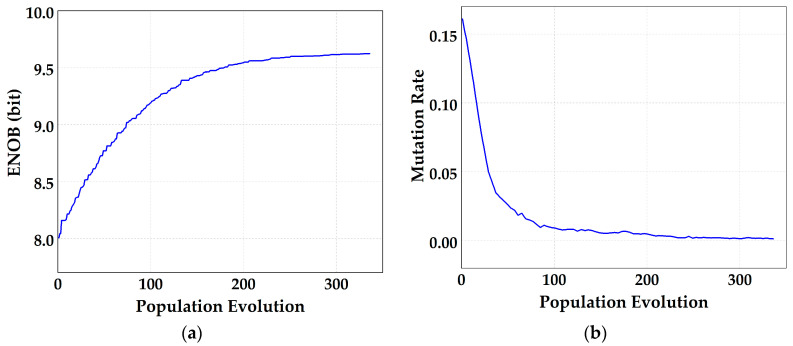
The trend of (**a**) ENOB as the fitness function with population evolution and (**b**) mutation rate with population evolution.

The measured differential non-linearity (DNL) and integral non-linearity (INL) are −0.57/+0.59 LSB and −3.67/+3.89 LSB before calibration, which are shown in Figure 10. Figure 11a shows the ADC’s spectral performance without foreground calibration at 25 °C. The SFDR is 66.7 dB and SNDR is 49.9 dB with a 20 MHz input signal before calibration. With the proposed calibration of capacitor mismatch and static IGE based on GA at this input signal, the SFDR/SNDR are improved by 10.8/9.7 dB compared with the condition before calibration, and the spectrum has also been greatly improved, as shown in Figure 11b. The DNL and INL are also improved to −0.45/+0.44 LSB and −1.81/+1.81 LSB with calibration, as shown in Figure 12. Figure 13 shows the trend of ENOB as the fitness function with population evolution and the mutation rate with population evolution.

**Figure 14:**
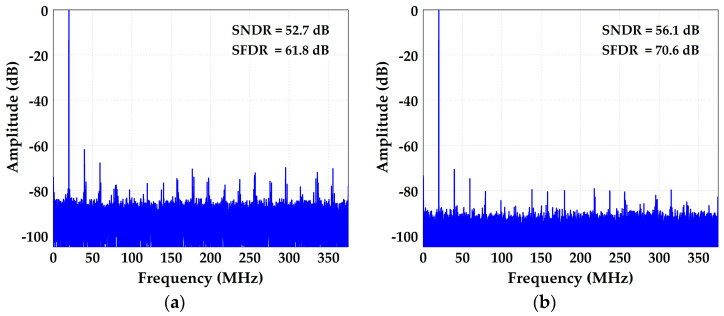
ADC spectral performance (**a**) without and (**b**) with background calibration at 85 °C.

When the temperature of the environment increases, the overall performance of the ADC will decrease. For example, the temperature rise will lead to a decrease in the open-loop gain of the operational amplifier, resulting in dynamic IGE. Figure 14a shows the ADC’s spectral performance without background calibration for dynamic IGE at 85 °C. When the background calibration is off, the SFDR is 61.8 dB and SNDR is 52.7 dB. When the background calibration is on, the SFDR is improved to 70.6 dB, and the SNDR is improved to 56.1 dB, as shown in Figure 14b. 

**Figure 15:**
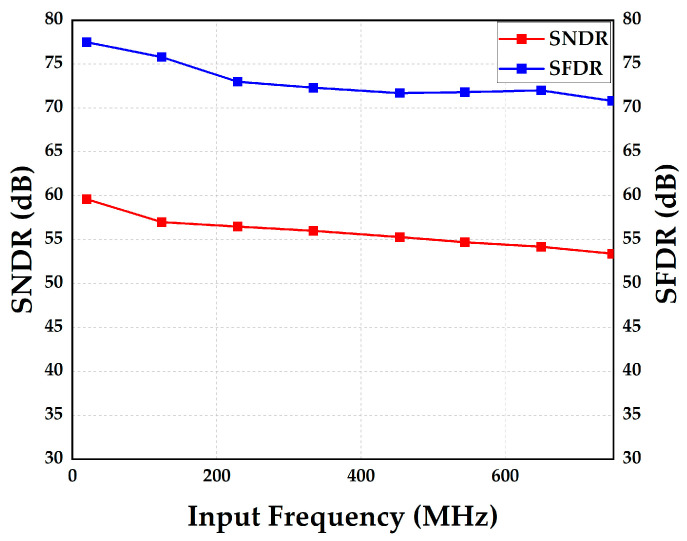
Measured SNDR/SFDR of this ADC versus frequency of the input signal with calibration at 750 MS/s.

Figure 15 shows the SFDR and SNDR of this ADC versus the frequency of the input signal with calibration at 750 MS/s. Table 1 lists the state-of-the-art calibrations applied to the pipelined ADC. Ref. [19] merges the interstage gain for each DAC signal path, similar to 1.5-bit dither-based calibration methods [18,35], instead of separately addressing IGE and capacitor mismatch. However, the path selection needs an additional shuffler controlled by a multi-bit pseudo-random sequence. The LMS-based background calibration is described in [36], but it requires an auxiliary slow but accurate ADC to statistically estimate and correct the IGE of the pipelined ADC. Compared to [19,36], modifications to analog circuits and overall calibration costs in this work and [37,38] are not significant. Ref. [37] introduces a low-power mixed-signal foreground calibration algorithm of a pipelined ADC using a digitally controlled reconfigurable switched capacitor MDAC gain controller that forces the front-end stage MDAC gain toward its ideal value to achieve the ideal ADC output linearity. Ref. [38] proposes a digital foreground calibration algorithm of a pipelined ADC, using the square wave signal as the input to remove the ADC’s non-linearities caused due to finite op-amp gain, capacitor mismatches and the effect of parasitic capacitance, but it cannot follow op-amp gain variations with voltage and temperature. This means it is not suitable for ultra-deep submicron technology, but only for deep submicron technology, such as 0.18 μm, so that the high intrinsic device gain can ensure a high op-amp gain over all the PVT corners. However, the algorithms in [37,38] have only been validated through simulation and have not been tested and verified. The proposed calibration in this work combines foreground calibration with background calibration in the digital domain to calibrate capacitor mismatch and IGE in MDAC, which is suitable for ultra-deep/deep submicron technology. Meanwhile, this can be achieved with only a small number of multipliers and adders, so the on-chip overhead is relatively small and has low cost.

## 5. Conclusions

In this paper, an M-D MDAC calibration method to calibrate errors caused by finite op-amp gain and capacitor mismatch in pipelined ADC is proposed. First, an M-D MDAC compensation model is proposed to achieve capacitor mismatch and static IGE compensation. First, a PN injection noise compensation coefficient, $\alpha_{PN}$, is designed in the M-D MDAC compensation model to finely classify and compensate the PN injection. This can also ensure dynamic IGE calibration is successful. Second, an M-D coefficient extraction scheme based on GA is proposed to avoid the coefficient falling into local traps in the compensation coefficient extraction, which can ensure the effectiveness of compensation. Since the M-D coefficients are not affected by temperature and voltage changes, they are calculated by the computer through MATLAB and written to the ADC internal registers for on-chip compensation. At the same time, we validated the above calibration algorithm based on a 12-bit 750 MS/s pipelined ADC. The measurement results show that the proposed calibration method increases the SNDR from 49.9 dB to 59.6 dB and the SFDR from 66.7 dB to 77.5 dB at 25 °C. When combined with background calibration at 85 °C, the SNDR and SFDR are improved by 3.4 dB and 8.8 dB, respectively. Due to the required M-D coefficients being calculated off-chip, the compensation and dynamic IGE calibration on-chip only require a small number of additional registers and multipliers, resulting in minimal overhead.

## Figures and Tables

**Table 1:** Comparison with state-of-the-art calibrations.

	[19]	[36]	[37]	[38]	This Work
Sampling Rate (MS/s)	100	250	100	100	750
Resolution (bits)	14	16	11	12	12
SNDR (dB)	65	76.5	65.13	71.88	59.6
SFDR (dB)	85	98	74.59	89.89	77.5
Fore/Back	Back	Back	Fore	Fore	Fore + Back
Domain	Digital	Analog + Digital	Analog + Digital	Analog + Digital	Digital
Sample	2^28^	/	/	/	2^26^
Overhead	Large	Large	Medium	Medium	Small

## Data Availability

Not applicable.
